# N-salicyloyl tryptamine derivative ameliorates spermatogenic dysfunction in obese mice by attenuating insulin resistance, enhancing Sertoli cell glycolysis, and inhibiting apoptosis: an *in vivo* and *in vitro* study

**DOI:** 10.3389/fcell.2026.1774188

**Published:** 2026-06-09

**Authors:** Zhenhui Fu, Yongyi Pan, Qiumei Huang, Hui Wu, Yunqian Cai, Changlei Yang, Xiaocan Lei, Zhen Wang, Fenglian Yang, Linlin Hu

**Affiliations:** 1 Key Laboratory of Research on Clinical Molecular Diagnosis for High Incidence Diseases in Western Guangxi, Department of Obstetrics and Gynecology, Department of reproductive medicine Center, Affiliated Hospital of Youjiang Medical University for Nationalities, Baise, Guangxi, China; 2 Institute of Clinical Anatomy & Reproductive Medicine Department of Histology and Embryology Hengyang Medical School University of South China, Hengyang, Hunan, China; 3 School of Pharmaceutical Science, School of Basic Medicine, Hengyang Medical School, University of South China, Hengyang, Hunan, China; 4 Guangxi Key Laboratory of Artificial Intelligence for Genetic Diseases of Long-dwelling Nationalities, Baise, Guangxi, China; 5 Guangxi Database Construction and Application Engineering Research Center for Intracorporal Pharmacochemistry of TCM, Youjiang Medical University for Nationalities, Baise, China

**Keywords:** glycolysis, insulin resistance, N-salicyloyl tryptamine, obesity, Sertoli cells, spermatogenic function

## Abstract

**Background:**

Obesity impairs male reproductive function, yet effective protective strategies are limited. Compound 51 is a novel N-salicyloyl tryptamine derivative whose role in spermatogenic function is unknown. This study aimed to determine whether it protects against obesity-induced spermatogenic dysfunction.

**Methods:**

Employing a combined *in vivo* and *in vitro* approach, we first evaluated compound 51 in a high-fat diet-induced obese mouse model, assessing systemic metabolism, reproductive phenotype, and molecular changes in testes. To investigate the underlying cellular mechanisms suggested by the *in vivo* findings, parallel experiments were conducted in palmitic acid-injured TM4 Sertoli cells, focusing on insulin signaling and glycolytic function.

**Results:**

*In vivo*, compound 51 administration significantly improved body weight, semen quality, testicular/epididymal morphology, and hormone levels (testosterone, follicle-stimulating hormone, inhibin B). It dose-dependently modulated apoptosis markers (downregulated Bax, upregulated Bcl-2/PCNA), enhanced testicular insulin sensitivity (reduced insulin-like growth factor 1 (IGF1) and elevated its receptor IGF1R), and increased glycolytic enzyme expression (hexokinase 2, pyruvate kinase M2, lactate dehydrogenase A). *In vitro*, compound 51 promoted proliferation, improved insulin sensitivity, and enhanced glycolytic activity in PA-injured TM4 cells, thereby ameliorating Sertoli cell dysfunction.

**Conclusion:**

Compound 51 ameliorates obesity-induced spermatogenic dysfunction by enhancing testicular insulin sensitivity and promoting glycolytic flux in Sertoli cells.

## Introduction

Obesity has evolved into a global epidemic and significantly increases the risk of cardiovascular disease, type 2 diabetes, and various cancers, thereby shortening life expectancy (Hui et al., 2025; Han, 2024; Blüher, 2019), but is also widely recognized as a key factor contributing to male infertility (Pereira et al., 2025; Shukur et al., 2026). Both clinical and experimental studies have shown that obesity induced by a high-fat diet (HFD) leads to testicular dysfunction, manifested as abnormal testicular morphology, reduced sperm count and motility, increased sperm abnormalities, and altered sex hormone levels (Taha et al., 2019; Chen et al., 2025). However, the underlying molecular mechanisms remain to be fully elucidated.

Obesity is a major contributor to insulin resistance (Arneth, 2024; Buscemi et al., 2024). Notably, insulin resistance has been confirmed to be closely associated with reduced male fertility and reproductive dysfunction (Andlib et al., 2023). As the structural and nutritional pillars of the seminiferous epithelium, Sertoli cells metabolize glucose to lactate, providing essential energy substrates for developing germ cells (Gorga et al., 2023; Rindone et al., 2023). A substantial body of evidence confirms that insulin resistance can directly impair Sertoli cell glucose metabolism, thereby compromising spermatogenesis (Crisóstomo et al., 2018; Gan et al., 2022; Oghbaei et al., 2021). Sertoli cell glycolytic dysfunction leads to reduced lactate production, which is not only an energy source for germ cells but also a critical anti-apoptotic factor that inhibits germ cell death in a dose-dependent manner, playing an essential role in maintaining germ cell survival (Yang et al., 2022; Bustamante-Marín et al., 2012; Erkkilä et al., 2002).

Apoptosis is a core mechanism for maintaining germ cell number balance and eliminating defective cells during testicular development and spermatogenesis (Hirai et al., 2004). Dysregulated apoptosis is highly correlated with obesity-induced male infertility (Dias et al., 2013), and HFD-fed mice exhibit significantly enhanced testicular germ cell apoptosis (Han et al., 2023). Furthermore, insulin plays a critical role in regulating the hypothalamic-pituitary-gonadal (HPG) axis and fertility (Oghbaei et al., 2021). The HPG axis operates through pulsatile release of gonadotropin-releasing hormone (GnRH) from the hypothalamus, which stimulates the anterior pituitary to secrete luteinizing hormone (LH) and follicle-stimulating hormone (FSH). These hormones act on Leydig cells and Sertoli cells to promote steroidogenesis and spermatogenesis (Ivell et al., 2014). At the Leydig cell level, insulin resistance impairs the expression of key steroidogenic proteins (such as StAR and 3β-HSD), leading to reduced testosterone production and further exacerbating spermatogenic impairment (Zhang et al., 2017a). Concurrently, insulin resistance can impair central HPG axis function, reducing secretion of GnRH, LH, FSH, and testosterone (Andlib et al., 2023).

Previous studies have demonstrated that the parent N-salicyloyl tryptamine derivative L7 exhibits potent anti-neuroinflammatory and anti-apoptotic activities (Bai et al., 2021; Zhao et al., 2024; Wu et al., 2024). Based on these findings, we designed a novel N-salicyloyl tryptamine derivative, compound 51. Which integrates structural moieties of salicylic acid and melatonin. Both have reported metabolic and reproductive benefits (Ding et al., 2023; Hasan et al., 2023; Yeasmin and Choi, 2020; Rocha et al., 2014). Given the central roles of insulin resistance and glycolytic metabolism in Sertoli cell function and spermatogenesis, compound 51 provides a rational basis for its potential protective effects against HFD-induced testicular injury.

This study aimed to investigate whether compound 51 protects against HFD-induced testicular dysfunction in obese mice. We analyzed testicular expression of proliferation/apoptosis factors, insulin resistance markers, and glycolytic rate-limiting enzymes. Our findings may provide insights for therapeutic strategies against obesity-related male infertility.

## Materials and methods

### Preparation and identification of compound 51

All the chemical reagents used in this study were purchased from the commercial supplier energy chemical (Shanghai, China). The data were recorded using a nuclear magnetic resonance (NMR) spectrometer (Bruker AVANCE500, Germany) and a high-performance liquid chromatography (HPLC) instrument (Shimzdcu, Japan).

### Method for compound 51

Step a: 5-Methoxytryptamine (2.0 g, 9.12 mmol) and 4-methylsalicylic acid (1.45 g, 8.29 mmol), EDCI (1.75 g, 9.12 mmol), and HOBt (1.23 g, 9.12 mmol) were separately added to a DCM (30 mL) solution. Triethylamine (1.4 mL, 9.95 mmol) was then added at room temperature and the mixture was stirred for 2 h. The reaction was monitored by TLC for 5-methoxytryptamine (eluent: PE/EA = 1:2) and 4-methylsalicylic acid (eluent: DCM/MeOH = 2:1), and no starting materials remained. The reaction was quenched with water and extracted three times with dichloromethane. The product was purified by column chromatography (PE/EA = 1:1) to yield compound 2 with a yield of 75%.

Step b: Intermediate 2 (2.0 g, 5.91 mmol) was dissolved in dichloromethane (DCM, 50 mL). Boron tribromide (BBr_3_, 2.8 mL, 29.55 mmol) was added at −78 °C. After stirring for 0.5 h, the reaction mixture was allowed to warm to room temperature and stirred for an additional 2 h. The reaction was monitored by TLC (eluent: PE/EA = 1:2), with a small amount of starting material remaining. The reaction was quenched with saturated NaHCO_3_ solution and extracted three times with dichloromethane. The product was purified by column chromatography (PE/EA = 1:1) to yield compound 51 with a yield of 36%.

### Spectral analysis of the compound 51

*hydroxy-N-(2-(5-hydroxy-1H-indol-3-yl)ethyl)-4-methylbenzamide* (Jia et al., 2018).White solid (36% yield). ^1^H NMR (500 MHz, CD_3_OD) δ 7.58 (d, *J* = 8.0 Hz, 1H), 7.16 (d, *J* = 8.6 Hz, 1H), 7.03 (s, 1H), 6.99 (s, 1H), 6.72–6.64 (m, 3H), 3.64 (t, *J* = 7.5 Hz, 2H), 2.98 (t, *J* = 7.5 Hz, 2H), 2.29 (s, 3H). ^13^C NMR (126 MHz, CD_3_OD) δ 171.0, 161.2, 151.1, 145.8, 133.1, 129.4, 128.7, 124.3, 121.1, 118.6, 114.4, 112.7, 112.5, 112.4, 103.6, 41.4, 26.4, 21.5.HRMS (TOF ESI^+^): m/z calcd. For C_19_H_19_NO_3_ [M + H]^+^, 311.1380; found, 311.1383; HPLC purity: 95.20%

### Experimental animals and diet

This study utilized 50 male C57BL/6J mice at 3 weeks of age, which were of SPF grade and provided by Hunan Slack Jingda Laboratory Animal Co., Ltd (Certificate No. SCXK (Xiang) 2021-0002, Changsha, China). The mice were acclimated for 1 week on a standard diet in a temperature-controlled environment (20 °C–23 °C) with a 12-h light/dark cycle. After the acclimation period, the mice were divided into two groups and maintained for 12 weeks: the control group (n = 10) continued on the standard diet, while the HFD group (n = 40) was fed an HFD (60% kcal from fat, Cat# D12492, Beijing Keao Feed Co., Beijing, China) (Huang et al., 2026). After 12 weeks, the HFD group was further randomly divided into four subgroups (n = 10 each) and maintained for an additional 8 weeks as follows: HFD continuation group, HFD +25 mg/kg compound 51 group, HFD +50 mg/kg compound 51 group (doses selected based on previous studies of structurally similar N-salicyloyl tryptamine derivatives (Zhao et al., 2025)), and HFD +60 mg/kg orlistat group (positive control, dose based on previous studies (Xu et al., 2023)). Throughout the experiment, mice had free access to food and water. Body weight was measured and recorded at a fixed time daily, along with 24-h food intake. After the feeding period, an oral glucose tolerance test (OGTT) was performed. Two days later, mice were anesthetized with 20% urethane (intraperitoneal injection). Blood samples were collected via cardiac puncture, followed by euthanasia by cervical dislocation.

### Oral glucose tolerance test (OGTT)

OGTT was conducted as described in reference (Xu et al., 2018). After an overnight fast, baseline blood glucose levels were measured from the tail vein using a glucometer. Mice were then orally administered glucose at a dose of 2 g/kg. Blood glucose levels were measured at 30, 60, 90, and 120 min post-administration using a glucometer.

### Serum biochemical analysis

Serum was separated by centrifugation. Levels of triglycerides (TG), total cholesterol (TC), low-density lipoprotein (LDL), high-density lipoprotein (HDL), alanine aminotransferase (ALT), and aspartate aminotransferase (AST) were measured using commercial kits (Nanjing Jiancheng Bioengineering Institute, Nanjing, China) according to the manufacturer’s instructions. The kit catalog numbers were as follows: TG (A110-1-1), TC (A111-1-1), LDL (A113-1-1), HDL (A112-1-1), ALT (C009-2-1), and AST (C010-2-1).

### Detection of testosterone (T), FSH, inhibin B (INHB) and insulin levels

Serum levels of T, FSH, INHB, and insulin were measured using commercial ELISA kits (Sangon Biotech, Shanghai, China) according to the manufacturer’s instructions. Absorbance was read at 450 nm. Kit catalog numbers were as follows: T (Cat# D721374), FSH (Cat# D721074), INHB (Cat# D721100), and insulin (Cat# D721197).

### Epididymal sperm analysis

Epididymal sperm analysis was performed according to the method described previously (Luo et al., 2023). The caudal epididymis was minced in 1.5 mL of physiological saline and incubated at 37 °C for 20 min. Sperm samples were then transferred to a Makler counting chamber (Sefi-Medical Instruments, Ltd., Israel) and examined under a microscope at ×20 magnification. For morphology assessment, 300 sperm per slide were stained with eosin, and the proportion of abnormal sperm was calculated.

### Morphological analysis of liver, adipose tissue, testes, and epididymis

Liver, adipose tissue, testis, and epididymis were fixed overnight in 4% paraformaldehyde, embedded in paraffin, and sectioned at 4 μm thickness. Sections were stained with hematoxylin and eosin (H&E) and imaged under an optical microscope. For quantitative analysis of seminiferous tubules, six mice per group were randomly selected (n = 6). From each mouse, five non-serial testicular sections were examined. In each section, ten randomly selected round or nearly round seminiferous tubule cross-sections were captured at ×400 magnification. The diameter and cross-sectional area of each tubule were measured using ImageJ software (National Institutes of Health, Bethesda, MD, United States of America). Prior to measurement, the image scale was calibrated using a micrometer slide image to convert pixels to micrometers. The basement membrane of each selected tubule was manually traced, and the software automatically calculated the diameter and area (Figueiró et al., 2017). All measurements were performed by two investigators who were blinded to the experimental groups.

### Cell culture

TM4 mouse Sertoli cells were obtained from the American Type Culture Collection (ATCC) via Cellcook Biotech Co., Ltd. (Guangzhou, China). The cells were cultured in DMEM/F-12 medium supplemented with 5% fetal bovine serum and 2.5% horse serum at 37 °C in a 5% CO_2_ humidified atmosphere and passaged every 1–2 days (Huang et al., 2026).

### Cell viability assay

According to a previously described method (Zhang et al., 2025). TM4 cells were seeded in 96-well plates and treated with various concentrations of compound 51. After 24 h, the medium was replaced with fresh medium containing 10% CCK-8 solution (Cat# BS350B, Biosharp, China) and incubated for 1–4 h. Absorbance was measured at 450 nm using a microplate reader.

### Measurement of lactate and pyruvate production

Lactate (Cat# A019-2-1) and pyruvate (Cat# A081-1-1) levels in TM4 cells were measured using kits from Nanjing Jiancheng Bioengineering Institute (Nanjing, China) according to the manufacturer’s instructions. Absorbance was read at 530 nm (lactate) and 505 nm (pyruvate).

### Immunohistochemistry analysis

Immunohistochemistry was performed following the protocol described in our previous study (Wang et al., 2023). Briefly, 4-μm paraffin sections were dewaxed, subjected to antigen retrieval in citrate buffer (10 mM, pH 6.0) by microwave treatment for 15 min, and treated with 3% H_2_O_2_ for 30 min to block endogenous peroxidase. Sections were permeabilized with 1% Triton X-100 in PBST for 30 min at room temperature, then blocked with 5% BSA for 45 min. Subsequently, the sections were incubated overnight at 4 °C with primary antibodies against the following markers: Vimentin (1:2000, Cat# A19607, ABclonal, China), PCNA (1:4000, Cat# 10205-2-AP, Proteintech, China),pyruvate kinase M2 (PKM2) (1:200, Cat# 15822-1-AP, Proteintech, China), hexokinase 2 (HK2) (1:1000, Cat# 22029-1-AP, Proteintech, China), lactate dehydrogenase A (LDHA) (1:100, Cat# 19987-1-AP, Proteintech, China), IGF1 (1:100, Cat# A11985, ABclonal, China) and IGF1R (1:100, Cat# PK65966, Abmart, China). After washing with PBST, the sections were incubated with Biotin-conjugated Goat Anti-Rabbit IgG (H + L) (1:500, Cat# SA00004-2, Proteintech, China) at room temperature and 37 °C for 45 min, followed by HRP-conjugated Streptavidin (1:200, Cat# SA00001-0, Proteintech, China) at 37 °C for 45 min. Immunoreactivity was visualized using DAB (3,3′-diaminobenzidine) solution. The reaction was stopped by discarding the solution and rinsing with PBST when color development was observed. Sections were counterstained with hematoxylin for 5 min, differentiated in 1% hydrochloric acid alcohol for 1 s, and rinsed with tap water. PBST served as a negative control. Stained sections were imaged under an optical microscope (BX43, Olympus, United States of America). Positive staining was defined as brown-yellow granules in the cytoplasm or nucleus and evaluated qualitatively.

### Quantitative real-time polymerase chain reaction (qRT-PCR) assay

According to a previously described method (Dang et al., 2026). Total RNA was extracted from mouse testicular tissue and TM4 cells using the AG RNAex Pro RNA extraction kit (Aicore Biotech, China). RNA (1 μg) was reverse transcribed into cDNA using the HiScript IV All-in-One Ultra RT SuperMix for qPCR kit (Vazyme, China) according to the manufacturer’s instructions. GAPDH was used as an internal reference for relative mRNA expression levels. Real-time PCR analysis was performed using the SYBR Green Pro Taq HS premix (with ROX, Cat# AG11718, Aicore Biotech, China) on the QuantStudio three real-time PCR system (Thermo Fisher Scientific, United States of America). Primers are listed in Table 1. The 2^-ΔΔCt method was used to calculate mRNA expression levels.

**TABLE 1:** Primers sequences used as target and reference genes used in qRT-PCR reactions.

Gene	Primer sequence (5′-3′)
Mus HK2	F: TGCCTGCTCCATCGGT R: TGGTAGAGATACTGGTCAACCTTC
Mus PKM2	F: GTGCCGCCTGGACATTGACTC R: TTCAGCCGAGCCACATTCATTCC
Mus LDHA	F: GGAGGGCAGCTTTCTAACCA R: GGACTTTGAATCTTTTGAGACCTTG
Mus IGF1	F: TGGATGCTCTTCAGTTCGTG R: GTCTTGGGCATGTCAGTGTG
Mus IGF1R	F: GCTTCGTTATCCACGACGATG R: GAATGGCGGATCTTCACGTAG
Mus PCNA	F: CCTGTGCAAAGAATGGGGTG R: TCTCTATGGTTACCGCCTCC
Mus BAX	F: TGCAGAGGATGATTGCTGAC R: GATCAGCTCGGGCACTTTAG
Mus Bcl-2	F: GACTGAGTACCTGAACCGGC R: AGTTCCACAAAGGCATCCCAG
Mus WT1	F: CCATCCGCAACCAAGGATAC R: CCATGGGGTCCTCGTGTTTG
Mus GATA4	F: ATGCCTGTGGCCTCTATCAC R: GGTGGTGGTAGTCTGGCAGT
Mus Vimentin	F: TTCTCTGGCACGTCTTGACC R: CTCCAGGGACTCGTTAGTGC

### Western blot (WB) analysis

As described previously (Xue et al., 2025). Proteins were extracted from mouse testicular tissues and TM4 cells using RIPA lysis buffer (Cat# P0013, Beyotime, China). Lysates were centrifuged at 12,000 rpm for 20 min, and the supernatant was collected. Protein concentration was determined using the BCA method. Equal amounts of protein were separated by 10% SDS-PAGE and transferred to PVDF membranes (Merck Millipore, United States of America). The membranes were blocked with 5% skim milk in PBST for 2 h at room temperature, incubated with primary antibodies overnight at 4 °C, and further incubated with horseradish peroxidase (HRP)-labeled goat anti-rabbit IgG (H + L) (1:5000, Cat# SA00001-1, Proteintech, China) secondary antibodies for 2 h. The membranes were then developed using an ultra-sensitive ECL chemiluminescence detection kit (Cat# PK10003, Proteintech, China) and visualized using the Tanon-5500 Chemiluminescence Imaging System (Tanon Science & Technology, Shanghai, China). Finally, band intensities were quantitatively analyzed using ImageJ software. The primary antibodies used for Western blotting included: β-Tubulin (1:5000, Cat# A12289, ABclonal, China), PKM2 (1:2000, Cat# 15822-1-AP, Proteintech, China), HK2 (1:1000, Cat# 22029-1-AP, Proteintech, China), LDHA (1:2000, Cat# 19987-1-AP, Proteintech, China), IGF1 (1:15000, Cat# A11985, ABclonal, China) and IGF1R (1:2000, Cat# PK65966, Abmart, China).

### Transcriptome sequencing and bioinformatic analysis

The RNA sequencing of testicular tissue was performed by Shanghai Biotech Co, Ltd. Total RNA was extracted using Trizol reagent (Invitrogen Life Technologies) and its concentration and purity were measured using a NanoDrop spectrophotometer (Thermo Fisher Scientific). For each sample, sequencing libraries were generated from 3 μg of RNA. The library fragments were purified using the AMPure XP system (Beckman Coulter, Brea, United States of America), followed by selective enrichment using an Illumina PCR primer mix. The products were purified using the AMPure XP system and quantified on a Bioanalyzer 2100 system using an Agilent High Sensitivity DNA kit (Agilent). Subsequently, the sequencing libraries were sequenced on a NovaSeq Xplus platform (Illumina). The DESeq2 R package (version 1.10.1) was used to compare gene expression levels between the two groups. Genes with an adjusted p value <0.05 by DESeq2 were classified as differentially expressed genes (DEGs). To determine the enrichment of Kyoto Encyclopedia of Genes and Genomes (KEGG) pathways in each group, we performed KEGG pathway enrichment analysis using the KEGG database (http://www.genome.jp/kegg/) and determined the statistical enrichment of differentially expressed genes using the cluster Profiler R package.

### Quantification and statistical analysis

All data distribution analyses were conducted using GraphPad Prism 8 software (GraphPad Software, CA, United States of America). Throughout the analysis, we thoroughly assessed both dependent and independent data that conformed to a normal distribution. Data are presented as the mean ± standard deviation (Mean ± SD). The normality of the distribution was tested using the Kolmogorov-Smirnov test. To determine significant differences among various treatment groups, we initially performed a one-way analysis of variance (One-Way ANOVA), followed by a Tukey’s post hoc test for further multiple comparisons analysis. These statistical methods were uniformly applied to all datasets, including both *in vivo* and *in vitro* experiments. A p value of <0.05 was considered to indicate statistical significance.

## Results

### Preparation and toxicity evaluation of compound 51

**FIGURE 1:**
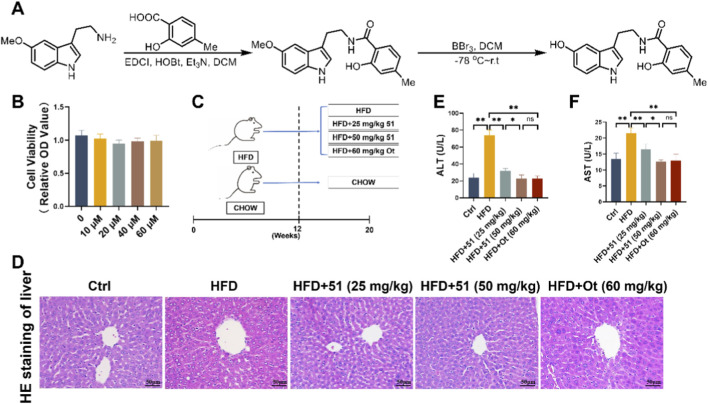
Design, toxicity validation, and obese mouse model construction of compound 51. **(A)** Synthetic route of compound 51. **(B)** Cell viability after treatment with different concentrations of compound 51 (n = 3). **(C)** Schematic diagram of the obese mouse model construction. **(D)** Representative images of H&E-stained liver tissue (scale bar = 50 μm). **(E)** Serum ALT level (n = 8). **(F)** Serum AST level (n = 8). ns, not significant; *p < 0.05; **p < 0.01. 51, compound 51; ALT, alanine aminotransferase; AST, aspartate aminotransferase; CHOW, standard chow diet; HFD, high-fat diet; Ot, orlistat.

Compound 51 was synthesized via a condensation reaction and demethylation with boron tribromide (Figure 1A). Its structure was characterized by 1H NMR, 13C NMR, and HRMS, and its purity was confirmed to be over 95% by HPLC analysis. Additionally, the cytotoxicity of compound 51 was assessed using the CCK8 assay, which showed that compound 51 had no significant effect on the viability of TM4 cells at various concentrations (Figure 1B). To investigate the *in vivo* effects of compound 51, we established a HFD-induced obese mouse model according to the experimental design illustrated in (Figure 1C). We then evaluated the safety of the selected doses by examining liver histology and serum biochemical markers. As shown in Figure 1D, HFD feeding induced notable hepatic histopathological changes, which were ameliorated by compound 51 treatment in a dose-dependent manner. Consistently, the elevated serum levels of ALT and AST in HFD-fed mice were significantly reduced by compound 51 in a dose-dependent manner (Figures 1E,F). Orlistat treatment also attenuated these abnormalities. These findings confirm the hepatoprotective effect of compound 51 and validate the safety of the selected doses for subsequent efficacy studies.

### Compound 51 improves body weight and lipid metabolic disorders in HFD-fed obese mice

**FIGURE 2:**
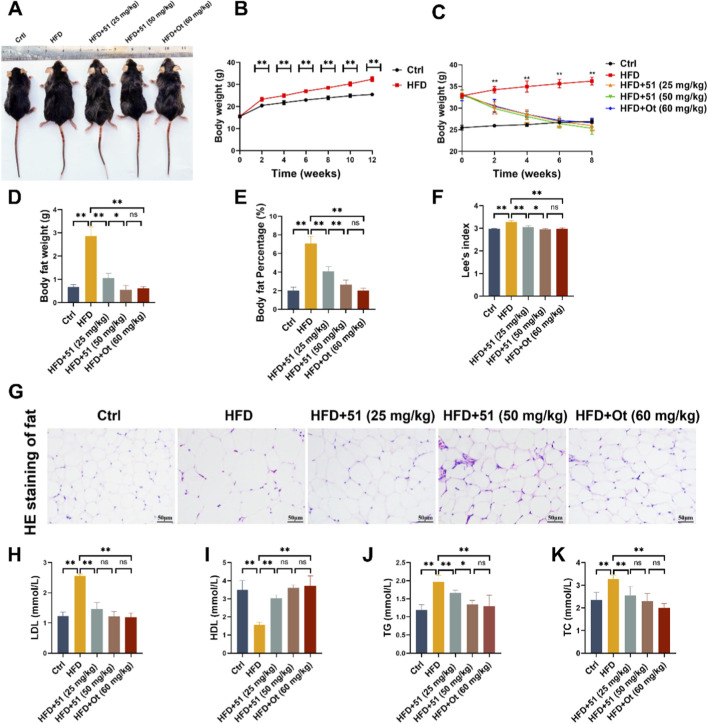
The effects of compound 51 intervention on lipid metabolism in obese mice. **(A)** Photographs of mouse body sizes. **(B)** Changes in body weight of mice in different dietary groups (n = 10). **(C)** Changes in body weight of mice under different intervention conditions (n = 10). **(D)** Fat weight (n = 10). **(E)** Percentage of body fat (n = 10). **(F)** Lee’s index (n = 10). **(G)** H&E staining of adipose tissue (scale bar = 50 μm). **(H)** Serum LDL (n = 6) level. **(I)** Serum HDL level (n = 6). **(J)** Serum TG (n = 6) level. **(K)** Serum TC level (n = 6). ns, not significant; *p < 0.05; **p < 0.01. 51, compound 51; HFD, high-fat diet; Ot, orlistat.

Compared to the control group on a regular diet, the HFD group exhibited a significant increase in body weight (Figures 2A,B), after 8 weeks of intervention with compound 51 (at doses of 25 and 50 mg/kg) or orlistat, weight gain was significantly suppressed (Figure 2C). Furthermore, compared with the HFD group, compound 51 intervention reduced fat weight, body fat percentage, Lee’s index, and adipocyte size (Figures 2D–G), and improved serum levels of triglycerides, cholesterol, low-density lipoprotein, and high-density lipoprotein (Figures 2H–K). The results were dose-dependent, with the parameters nearly returning to the levels of the orlistat group at a dose of 50 mg/kg.

### Compound 51 ameliorates the testis volume, epididymis volume, semen quality, morphology of testis and epididymis, and levels of sex hormones in obese mice fed HFD

**FIGURE 3:**
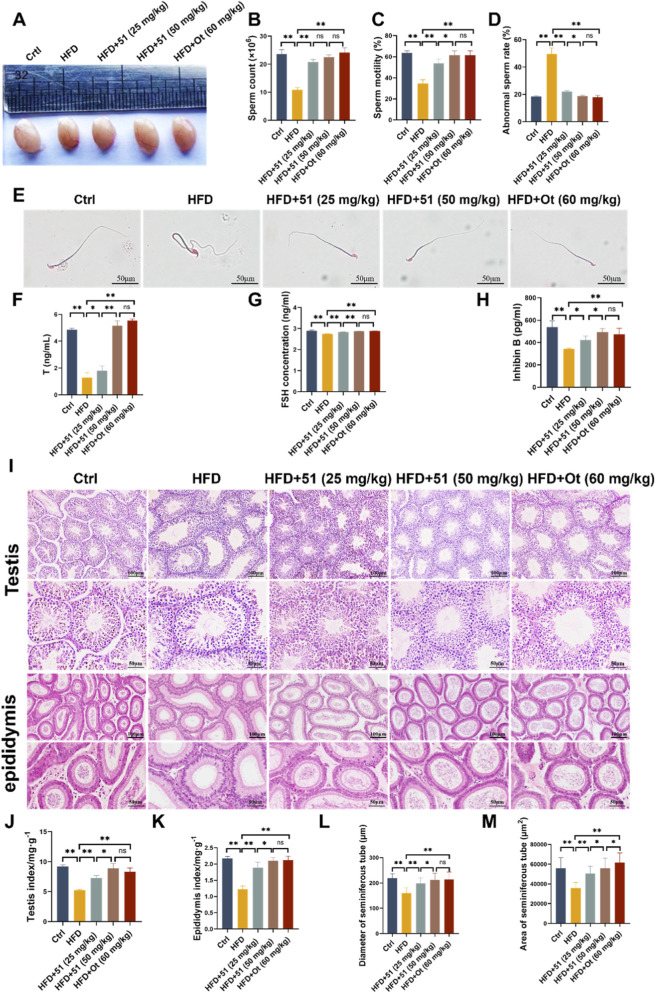
Effects of compound 51 intervention on spermatogenic function in the testes of obese mice. **(A)** Representative images of testis morphology. **(B)** Total sperm count (n = 8). **(C)** Sperm motility (n = 8). **(D)** Percentage of abnormal sperm (n = 8). **(E)** Representative images of sperm morphology (scale bar = 50 μm). **(F)** Serum testosterone (T) level (n = 6). **(G)** Serum FSH level (n = 6). **(H)** Serum INHB level (n = 6). **(I)** H&E staining of testicular and epididymal tissues (scale bar = 50–100 μm). **(J)** Testicular index (n = 8). **(K)** Epididymal index (n = 8). **(L)** Diameter of seminiferous tubules. **(M)** Cross-sectional area of seminiferous tubules. ns, not significant; *p < 0.05; **p < 0.01. 51, compound 51; FSH, follicle-stimulating hormone; HFD, high-fat diet; INHB, inhibin B; Ot, orlistat; T, testosterone.

Compared with the control group, the testis volume of obese mice was significantly reduced, sperm count decreased, sperm motility declined, and the proportion of abnormal sperm significantly increased. After 8 weeks of intervention with compound 51 (25 or 50 mg/kg) or orlistat, these parameters were significantly improved in a dose-dependent manner, and the effect of the 50 mg/kg compound 51 group was comparable to that of the orlistat group, (Figures 3A–D).Sperm morphology analysis revealed that control mice had regularly shaped sperm, whereas sperm from obese mice exhibited severe morphological abnormalities. After treatment with 25 mg/kg compound 51, some sperm showed improved morphology, although mild deformities such as slightly folded tails persisted. However, after treatment with 50 mg/kg compound 51 or orlistat, sperm morphology essentially returned to normal (Figure 3E). Serum hormone levels, including testosterone, FSH, and inhibin B (INHB), were within normal ranges in control mice but were significantly decreased in HFD-fed mice. After compound 51 intervention, these hormone levels increased in a dose-dependent manner. The 25 mg/kg dose elicited moderate elevations, while the 50 mg/kg dose produced more substantial increases, with levels approaching those of the control group. Significant elevations were also observed in the orlistat-treated group (Figures 3F–H). The pathological results of the testes and epididymis showed that in the control group mice, the germ cells in the seminiferous tubules of the testes were tightly arranged, with different stages of differentiated germ cells neatly lined up within the seminiferous tubules. In contrast, the seminiferous tubules of the obese mice exhibited atrophy, with sparse and disordered arrangement of germ cells. Additionally, sperm were hardly visible in the lumen of the epididymal tubules of the obese mice. After 8 weeks of intervention with compound 51 these pathological alterations were dose-dependently alleviated, with the 50 mg/kg dose showing the most pronounced recovery. Orlistat treatment also partially reversed these abnormalities (Figure 3I). Compared with the control group, the testis index and epididymis index of obese mice were significantly decreased, and the diameter and area of the seminiferous tubules were reduced. However, these parameters were significantly restored following compound 51 intervention in a dose-dependent manner: the 25 mg/kg dose produced moderate improvements, whereas the 50 mg/kg dose resulted in near-complete restoration. Significant improvements were also observed in the orlistat-treated group (Figures 3J–M).

### Transcriptomic analysis of the effects of compound 51 on the testes of obese mice

**FIGURE 4:**
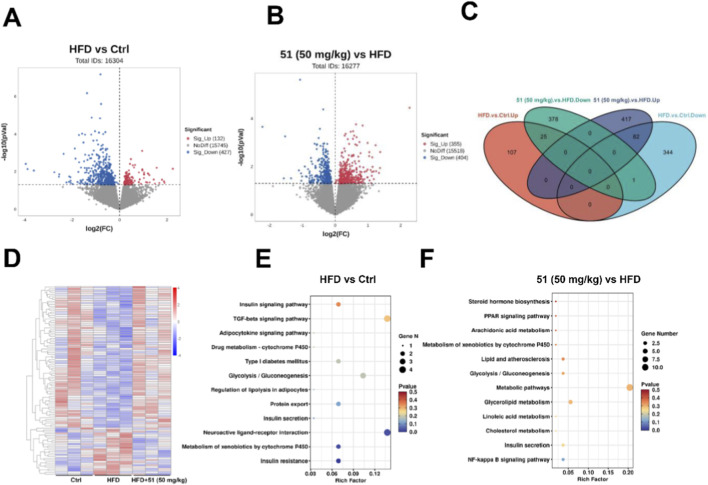
Effects of compound 51 intervention on the testicular transcriptome in obese mice. **(A)** Volcano plot of differentially expressed genes (DEGs) between the HFD group and the Ctrl group. **(B)** Volcano plot of DEGs between the 51 (50 mg/kg) group and the HFD group. **(C)** Venn diagram showing the overlap of DEGs. **(D)** Heatmap of differentially expressed genes across three sample groups. **(E)** Enrichment analysis plot between the HFD group and the Ctrl group **(F)** Enrichment analysis plot between the 51 (50 mg/kg) group and the HFD group. 51, compound 51; HFD, high-fat diet; Ot, orlistat.

To verify the effects of compound 51 on the testicular transcriptome of obese mice, we performed whole-transcriptome RNA sequencing (RNA-seq) on testicular tissue to explore its molecular mechanisms in reproductive damage induced by HFD in obese mice. Compared with the control group, the HFD group had 132 upregulated genes and 427 downregulated genes. Compared with the HFD group, the HFD +50 mg/kg compound 51 group had 355 upregulated genes and 404 downregulated genes (Figures 4A,B). Among these differentially expressed genes, 25 of the 132 upregulated genes and 82 of the 427 downregulated genes in the HFD group were significantly reversed in expression after intervention with 50 mg/kg compound 51 (Figure 4C). We conducted KEGG functional enrichment analysis and found that these differentially expressed genes were significantly enriched in signaling pathways such as glycolysis and insulin secretion (Figures 4D–F).

### Compound 51 improves insulin sensitivity in the testis of obese mice and reverses PA-induced insulin resistance in TM4 cells

**FIGURE 5:**
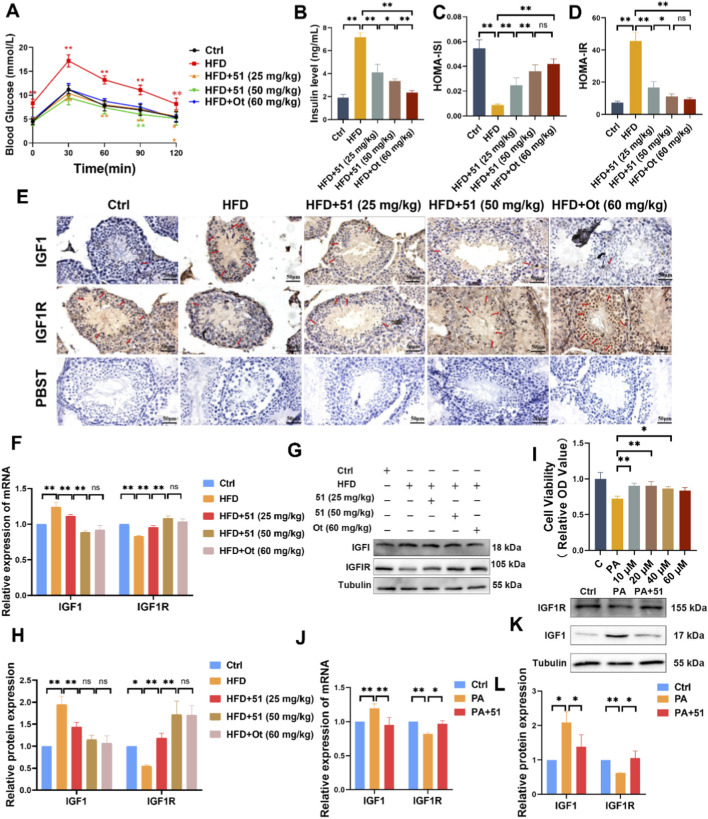
Compound 51 improves insulin sensitivity in the testis of obese mice and reverses PA-induced insulin resistance in TM4 cells. **(A)** Results of the OGTT in mice (n = 6). **(B)** Insulin levels (n = 6). **(C)** HOMA-ISI levels (n = 6). **(D)** HOMA-IR levels (n = 6). **(E)** Immunohistochemical analysis of IGF1 and IGF1R expression in the testes, with PBST as a negative control (scale bar = 50 μm). **(F)** qRT-PCR validation of IGF1 and IGF1R expression in the testes (n = 3). **(G)** Expression of IGF1 and IGF1R in the testes detected by Western blot analysis (n = 3). **(H)** Quantitative analysis of Western blot results using ImageJ software (Figure 5G). Protein levels were relatively quantified with tubulin as a reference (n = 3). **(I)** TM4 cells were treated with different concentrations of compound 51 and 0.4  mM PA for 24 h (n = 3). **(J)** qRT-PCR validation of IGF1 and IGF1R expression in PA-treated TM4 cells (n = 3). **(K)** Expression of IGF1 and IGF1R in PA-treated TM4 cells detected by Western blot analysis (n = 3). **(L)** Quantitative analysis of the Western blot results using ImageJ software (Figure 5K). Protein levels were relatively quantified with tubulin as a reference (n = 3). ns, not significant; *p < 0.05; **p < 0.01. 51, compound 51; HFD, high-fat diet; HOMA-IR, homeostasis model assessment of insulin resistance; HOMA-ISI, homeostasis model assessment of insulin sensitivity index; Ot, orlistat; PA, palmitic acid; qRT-PCR, quantitative real-time PCR.

Compound 51 enhanced insulin sensitivity in the testicular tissue of obese mice. In this study, we evaluated insulin sensitivity in the testicular tissue of the HFD induced obese mouse model. Compared with the control group, HFD-fed mice exhibited significantly impaired OGTT, decreased HOMA-ISI (insulin sensitivity index), and significantly elevated levels of fasting insulin and HOMA-IR (insulin resistance index). After intervention with compound 51, the levels of OGTT and HOMA-ISI in obese mice were significantly increased, while the levels of fasting insulin and HOMA-IR were significantly decreased (Figures 5A–D). Additionally, we assessed the impact of obesity on testicular IR by examining the expression of insulin-like growth factor 1 (IGF1) and its receptor (IGF1R). The results of immunohistochemical analysis showed that, compared with the control group, the expression of IGF1 was increased and the expression of IGF1R was decreased in the obese group. After intervention with compound 51, the expression of IGF1 decreased and the expression of IGF1R increased. (Figure 5E). qRT-PCR results showed that in obese mice, IGF1 expression levels were elevated, while IGF1R expression levels were reduced. After intervention with compound 51, the mRNA expression levels of IGF1 and IGF1R were significantly restored to normal (Figure 5F). Western blot analysis further confirmed the immunohistochemical analysis and qRT-PCR results, with increased IGF1 expression and decreased IGF1R expression in the obese group. These expression levels were improved after intervention with compound 51 (Figures 5G,H). All the above results demonstrated the dose-dependent ameliorative effects of compound 51, with the therapeutic effect of the 50 mg/kg dose group being comparable to that of the orlistat group. To further investigate the direct effects of compound 51 on Sertoli cells under lipotoxic conditions, we established an *in vitro* model using TM4 cells treated with 0.4 mM palmitic acid (PA), a concentration previously shown to reduce cell viability by more than 50% (Luo et al., 2023). Compound 51 treatment significantly restored PA-induced reduction in cell viability, with optimal effects observed at 20 μM (Figure 5I). Consistent with the *in vivo* findings, PA treatment significantly decreased IGF1R expression and increased IGF1 expression at both the mRNA and protein levels, whereas co-treatment with compound 51 effectively reversed these alterations (Figures 5J–L).

### Compound 51 promotes glycolytic metabolism and suppresses apoptosis in the testis of obese mice and in PA-treated TM4 cells

**FIGURE 6:**
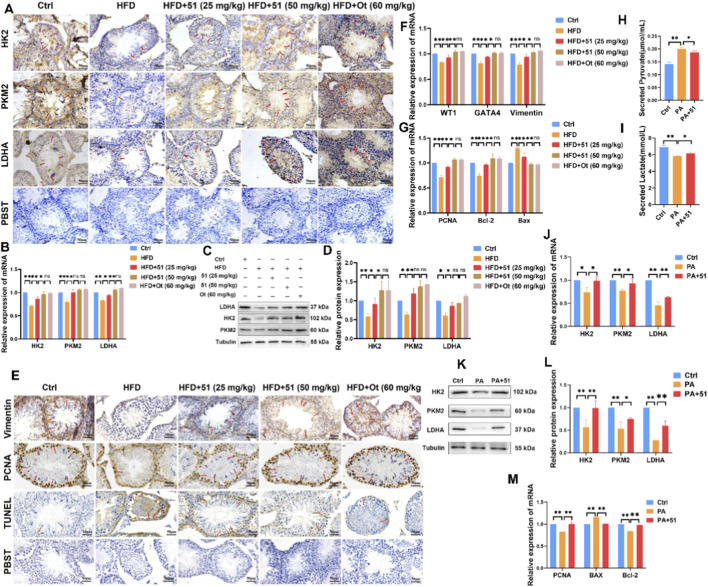
Compound 51 promotes glycolytic metabolism and suppresses apoptosis in the testis of obese mice and in PA-treated TM4 cells. **(A)** Immunohistochemical analysis of HK2, PKM2, and LDHA expression in the testes, with PBST serving as a negative control (scale bar = 50 μm). **(B)** qRT-PCR validation of HK2, PKM2, and LDHA expression in the testes (n = 3). **(C)** Detection of HK2, PKM2, and LDHA expression in the testes by Western blot analysis (n = 3). **(D)** Quantitative analysis of the Western blot results using ImageJ software (Figure 6C). Protein levels were relatively quantified with tubulin as a reference (n = 3). **(E)** Immunohistochemical analysis of Vimentin, PCNA and TUNEL expression in the testes, with PBST used as a negative control (scale bar = 50 μm). **(F)** qRT-PCR validation of WT1, GATA4, and Vimentin expression in the testes (n = 3). **(G)** PCNA, Bcl-2 and BAX mRNA expression in the testes (n = 3). **(H)** Pyruvate content in the culture medium of PA-treated TM4 cells (n = 3). **(I)** Lactate content in the culture medium of PA-treated TM4 cells (n = 3). **(J)** qRT-PCR validation of HK2, PKM2, and LDHA expression in PA-treated TM4 cells (n = 3). **(K)** Expression of HK2, PKM2, and LDHA in PA-treated TM4 cells detected by Western blot analysis (n = 3). **(L)** Quantitative analysis of the Western blot results using ImageJ software (Figure 6K). Protein levels were relatively quantified with tubulin as a reference (n = 3). **(M)** qRT-PCR validation of PCNA, BAX, and Bcl-2 expression in PA-treated TM4 cells (n = 3). ns, not significant; *p < 0.05; **p < 0.01. 51, compound 51; HFD, high-fat diet; Ot, orlistat; PA, palmitic acid; qRT-PCR, quantitative real-time PCR.

Immunohistochemical analysis results revealed differences in the expression of glycolytic rate-limiting enzyme genes between obese mice fed a HFD and control mice. Specifically, the positive cell expression of HK2, PKM2, and LDHA in the testicular tissue of obese mice was significantly lower than that of the control group. However, after treatment with compound 51, the number of positive cells for these glycolytic rate-limiting enzymes in the testes of obese mice significantly increased (Figure 6A). Further qRT-PCR analysis showed that the mRNA expression levels of HK2, PKM2, and LDHA were significantly reduced in obese mice. Treatment with compound 51 significantly improved the expression levels of these genes (Figure 6B). Western blot analysis also showed a similar trend (Figures 6C,D). The results were dose-dependent, with the high-dose treatment group showing results close to the orlistat group. To assess Sertoli cell function, we examined the expression of Vimentin, a structural marker of Sertoli cells, along with the functional markers GATA4 and WT1. Immunohistochemical analysis showed that Vimentin expression in the obese group was fragmented and scattered, with a significant reduction in Vimentin-positive cells compared with the control group, while compound 51 treatment restored Vimentin expression and organization (Figure 6E). qRT-PCR analysis further confirmed that the mRNA expression levels of Vimentin, GATA4, and WT1 were reduced in obese mice and were significantly restored following compound 51 treatment (Figure 6F). Obese mice also exhibited a significant reduction in PCNA-positive cells and a concurrent increase in TUNEL-positive cells, which was markedly attenuated by compound 51 treatment (Figure 6E). qRT-PCR analysis showed that Bax mRNA expression was elevated in obese mice, while PCNA and Bcl-2 mRNA levels were reduced, and compound 51 treatment effectively increased PCNA and Bcl-2 expression while decreasing Bax expression in a dose-dependent manner (Figure 6G). To further investigate the direct effects of compound 51 on Sertoli cells under lipotoxic conditions, we established an *in vitro* model using TM4 cells treated with 0.4 mM palmitic acid (PA). Consistent with the *in vivo* findings, PA treatment significantly reduced lactate production and increased pyruvate accumulation, whereas compound 51 co-treatment significantly increased lactate levels and reduced pyruvate levels (Figures 6H,I). qRT-PCR and Western blot analysis further revealed that PA treatment decreased the expression of HK2, PKM2, and LDHA at both mRNA and protein levels, which was effectively reversed by compound 51 (Figures 6J–L). Moreover, PA treatment significantly reduced PCNA and Bcl-2 expression while increasing Bax expression, and co-treatment with compound 51 significantly restored the expression of these factors (Figure 6M), corroborating the anti-apoptotic effects observed *in vivo*.

## Discussion

Obesity-induced male infertility is caused by the interplay of multiple factors, including systemic metabolic dysregulation, disruption of the HPG axis, and local testicular dysfunction (Mermer et al., 2018; Valsaraj et al., 2021; Leisegang et al., 2020). In recent years, obesity-induced inflammatory responses have also been implicated in male reproductive impairment (Zhang et al., 2017b). In this study, compound 51 reduced body weight, adiposity, and serum triglyceride and total cholesterol levels in obese mice. This systemic metabolic improvement may be attributed to its unique molecular structure. Studies have shown that salicylates exert anti-obesity and anti-inflammatory effects by inhibiting NF-κB-mediated chronic inflammation (Rocha et al., 2014), while the melatonin moiety protects against obesity-associated testicular dysfunction by upregulating PPAR-γ expression (Saidi et al., 2022) or stimulating the SIRT1/Nrf2/HO-1 pathway (Jan et al., 2024). Consistent with these findings, compound 51 intervention significantly improved metabolic parameters in obese mice, which may contribute to alleviating obesity-induced reproductive dysfunction.

Compound 51 improved also hormonal profiles. The intervention decreased serum insulin levels and increased testosterone and FSH levels. Obesity-associated insulin resistance is often accompanied by hyperinsulinemia, which inhibits hypothalamic Kisspeptin neuron activity, reduces pulsatile GnRH secretion, subsequently decreases LH and FSH release, and ultimately impairs Leydig cell steroidogenesis and spermatogenesis (Andlib et al., 2023; Fernandez et al., 2019). Clinical studies have confirmed that improving insulin sensitivity reverses hyperinsulinemia-induced testosterone reduction and impaired sperm quality (Montanino Oliva et al., 2016). Our study further extends this understanding: compound 51 not only restored insulin levels but also concomitantly increased FSH and testosterone levels, suggesting systemic reconstruction of HPG axis function.

Compound 51 restored Sertoli cell glycolysis. Sertoli cells play a critical role as “nurse cells” in spermatogenesis, converting glucose into lactate through highly active glycolysis. Lactate provides essential energy substrates for germ cells undergoing meiosis. It also acts as an anti-apoptotic signaling molecule to maintain germ cell survival (Hirai et al., 2004; Rotimi et al., 2024). In this study, HFD feeding led to downregulation of Sertoli cell marker genes (WT1, GATA4, Vimentin) and significant reduction in the expression of glycolytic rate-limiting enzymes (HK2, PKM2, LDHA), indicating impaired Sertoli cell function and metabolic failure. This finding is consistent with previous reports that HFD induces inhibition of Sertoli cell glycolysis, leading to insufficient lactate supply and subsequent spermatogenic dysfunction (Huang et al., 2026; Luo et al., 2023). Compound 51 intervention significantly restored all the above indicators.

Compound 51 reduced germ cell apoptosis. Dysregulated apoptosis is recognized as a key mechanism underlying obesity-induced male infertility (Dias et al., 2013). HFD-fed mice exhibited increased expression of the pro-apoptotic protein BAX, decreased expression of the anti-apoptotic protein Bcl-2 and proliferating cell nuclear antigen (PCNA), accompanied by pathological changes in the testis and epididymis as well as decreased semen quality, all of which were reversed by compound 51. This is consistent with numerous studies showing that HFD-induced obesity leads to increased germ cell apoptosis in testicular tissue (Luo et al., 2019; Jia et al., 2018), and dysfunction of the germ cell apoptosis mechanism can result in poor semen quality in ejaculated sperm (Li et al., 2023; Ji et al., 2009). By restoring lactate production via Sertoli cell glycolysis, compound 51 provides adequate energy to germ cells and inhibits apoptotic signals.

Compound 51 increased testosterone levels, providing insight into Leydig cell function. Studies have shown that Leydig cells produce testosterone, which acts on Sertoli cells to promote blood-testis barrier formation, completion of germ cell meiosis, and spermatogenesis (Ivell et al., 2014). The significant increase in serum testosterone levels strongly suggests restored Leydig cell function. As the primary energy metabolite secreted by Sertoli cells, lactate promotes testosterone synthesis by regulating steroidogenic enzymes (StAR, P450scc, 3β-HSD) (Rato et al., 2012; Lin et al., 2001; Chen et al., 2017). Therefore, the improvement in Sertoli cell function and the increase in lactate production following compound 51 treatment may also contribute to Leydig cell steroidogenesis. However, this study has certain limitations, as direct evidence of morphological changes in Leydig cells or expression of key steroidogenic enzymes is currently lacking. Therefore, although elevated serum testosterone levels indirectly suggest restoration of Leydig cell function, whether compound 51 directly acts on Leydig cells or exerts its effects indirectly through Sertoli cell-Leydig cell crosstalk requires further investigation. Future studies should examine the expression levels of key enzymes such as StAR, P450scc, and 3β-HSD, combined with ultrastructural observation of Leydig cells, to provide more direct evidence for Leydig cell functional recovery.

## Conclusion

In conclusion, this study demonstrates that compound 51 significantly alleviates high-fat diet-induced spermatogenic dysfunction by coordinately regulating insulin sensitivity and glycolysis in Sertoli cells. *In vivo*, compound 51 treatment improved insulin sensitivity in obese mice, restored testicular glycolytic activity, and reduced germ cell apoptosis. *In vitro*, compound 51 directly promoted lactate production in Sertoli cells, confirming its essential metabolic support role in spermatogenesis. These findings establish compound 51 as a promising candidate for the treatment of obesity-associated male infertility and provide mechanistic insights into the interplay between systemic insulin signaling and testicular metabolic homeostasis.

## Limitations of the study

Although the present study demonstrates that compound 51 ameliorates obesity-induced testicular dysfunction by restoring insulin sensitivity and promoting Sertoli cell glycolysis, several limitations should be acknowledged. First, although N-salicyloyl tryptamine derivatives have been previously reported to possess anti-inflammatory properties and compound 51 was rationally designed to incorporate the anti-inflammatory pharmacophore of salicylic acid, we did not directly assess the impact of compound 51 on inflammatory signaling within the testicular microenvironment. Given the well-established role of chronic inflammation in obesity-induced reproductive dysfunction, whether anti-inflammatory mechanisms contribute to the observed protective effects remains to be determined. Second, while we observed restoration of serum FSH, inhibin B, and testosterone levels in compound 51-treated obese mice, the neuroendocrine basis underlying these alterations warrants further investigation, particularly regarding the HPG axis. Finally, this study was conducted exclusively in a high-fat diet-induced obese mouse model. Although this model is highly relevant to human metabolic disease, validation of our findings using human Sertoli cell lines or testicular tissue samples from obese individuals would provide greater insight into the translational relevance for obesity-induced male infertility.

## Data Availability

The raw transcriptome data are available from the corresponding author upon reasonable request.
